# The development of ORACLe: a measure of an organisation’s capacity to engage in evidence-informed health policy

**DOI:** 10.1186/s12961-015-0069-9

**Published:** 2016-01-14

**Authors:** Steve R Makkar, Tari Turner, Anna Williamson, Jordan Louviere, Sally Redman, Abby Haynes, Sally Green, Sue Brennan

**Affiliations:** 1 The Sax Institute, Level 13, Building 10, 235 Jones Street, Ultimo, Sydney, NSW 2007 Australia; 2 World Vision Australia, 1 Vision Drive, Burwood East, Melbourne, Victoria 3151 Australia; 3 Institute for Choice, University of South Australia, Level 13, 140 Arthur Street, North Sydney, NSW 2060 Australia; 4 Sydney School of Public Health, University of Sydney, Edward Ford Building, Fisher Road, Sydney, NSW 2006 Australia; 5 School of Public Health and Preventive Medicine, Monash University, Level 6, The Alfred Centre, 99 Commercial Road, Melbourne, VIC 3004 Australia

**Keywords:** Assessment, Capacity, Discrete choice experiments, Evidence, Health policy, Knowledge translation, Measure, Organisation, Policymaker, Research, Research use

## Abstract

**Background:**

Evidence-informed policymaking is more likely if organisations have cultures that promote research use and invest in resources that facilitate staff engagement with research. Measures of organisations’ research use culture and capacity are needed to assess current capacity, identify opportunities for improvement, and examine the impact of capacity-building interventions. The aim of the current study was to develop a comprehensive system to measure and score organisations’ capacity to engage with and use research in policymaking, which we entitled ORACLe (Organisational Research Access, Culture, and Leadership).

**Method:**

We used a multifaceted approach to develop ORACLe. Firstly, we reviewed the available literature to identify key domains of organisational tools and systems that may facilitate research use by staff. We interviewed senior health policymakers to verify the relevance and applicability of these domains. This information was used to generate an interview schedule that focused on seven key domains of organisational capacity. The interview was pilot-tested within four Australian policy agencies. A discrete choice experiment (DCE) was then undertaken using an expert sample to establish the relative importance of these domains. This data was used to produce a scoring system for ORACLe.

**Results:**

The ORACLe interview was developed, comprised of 23 questions addressing seven domains of organisational capacity and tools that support research use, including (1) documented processes for policymaking; (2) leadership training; (3) staff training; (4) research resources (e.g. database access); and systems to (5) generate new research, (6) undertake evaluations, and (7) strengthen relationships with researchers. From the DCE data, a conditional logit model was estimated to calculate total scores that took into account the relative importance of the seven domains. The model indicated that our expert sample placed the greatest importance on domains (2), (3) and (4).

**Conclusion:**

We utilised qualitative and quantitative methods to develop a system to assess and score organisations’ capacity to engage with and apply research to policy. Our measure assesses a broad range of capacity domains and identifies the relative importance of these capacities. ORACLe data can be used by organisations keen to increase their use of evidence to identify areas for further development.

**Electronic supplementary material:**

The online version of this article (doi:10.1186/s12961-015-0069-9) contains supplementary material, which is available to authorized users.

## Background

There are a number of factors that bear influence on the formulation of health policy, including political pressures, stakeholder interests, media influence, resource availability, previous policies, and evidence from research [1-4]. However, there have been increased calls worldwide to strengthen the use of research evidence in the development, evaluation and implementation of policies [5-7]. Research is purported to provide reliable and valid evidence with which to inform decisions and formulate effective solutions in response to problems, and numerous studies have demonstrated associations between evidence-informed policies and improvements in health and spending [8]. Despite this, evidence suggests that, currently, many opportunities to use research to inform policymaking are missed [9-18].

There is growing recognition that an organisation’s technical capacity, climate and culture collectively affect employees’ work performance, adoption of and adherence to innovative and best work practices, and their expectations, attitudes, commitment, and value towards their work [19-23]. Indeed, evidence indicates that policymakers’ use of research can be improved if their organisations (1) have a receptive attitude and culture towards research use and (2) invest in resources that support research use capacity among staff (e.g. training programs, availability of research expertise, access to research databases, and other tools [24-28]). In light of this evidence, it is essential that valid and reliable measures of the capacity and culture of organisations to support the use of research in policymaking are developed. Such measures are necessary to enable agencies to understand whether there are opportunities for increasing their use of evidence, where these opportunities for improvement exist within the agency, what particular strategies could be implemented to improve the capacity of staff to better engage with and use research evidence in policymaking, and whether or not these capacity building strategies have been effective [29,30].

A small number of measures of organisational research use capacity are available, although they possess some key limitations. For example, (1) they are not specifically directed at policy agencies and evidence-informed policy [31]; (2) they measure barriers to research use rather organisational capacity to support research use [31]; (3) they lack reference to an explicit conceptual framework [31,32], thus failing to elucidate the influence of the wider policymaking context on organisational capacity and the potential impact of such capacity on policymakers’ engagement with and use of research [33,34]; (4) some aspects of organisational capacity are not addressed, such as the availability of tools to support leadership for research use [23] or mechanisms to commission and generate research to inform policy [28]; (5) the relative importance of different organisational tools and supports are not taken into account in these measures’ scoring systems, which has important implications for the practical utility of these measures for policy organisations; and finally (6) these measures are typically completed by staff members rather than the organisations’ leaders or executives. Consequently, what they measure are staff perceptions rather than the objective availability of tools within the organisation to support research use.

Given the likely critical importance of organisational capacity, and the dearth of suitable objective measures, we aimed to develop a comprehensive, valid, objective, and theory-based measure of organisational capacity to engage with and use research in policy development, which we entitled ORACLe (Organisational Research Access, Culture, and Leadership). ORACLe arose from a need to develop a comprehensive suite of measures to evaluate the impact of a multifaceted intervention program designed to increase organisations’ use of research in policy, entitled SPIRIT (Supporting Policy In health with Research: an Intervention Trial) [35]. Specifically, measures were developed to evaluate the impact of the SPIRIT intervention on organisations’ capacity to support research use (i.e. ORACLe), policymakers’ self-reported capacity, engagement with, and use of research (i.e. SEER (Seeking, Engaging, and Evaluating Research) [35]), and the use of research in the development of discrete policy documents (i.e. SAGE [35]).

As a measure of organisational capacity, ORACLe is grounded in the SPIRIT Action Framework [34], which postulates that the extent to which an organisation and staff have the capacity to engage with research directly mediates whether staff will effectively engage with and use research to inform policymaking. Because of the link between organisational capacity and staff capacity to engage with and use research to inform policymaking, ORACLe is designed to assess multiple aspects of organisational capacity, including the systems, supports, and tools organisations have in place to enable research use, and the value placed on research by the organisation. ORACLe is administered as a structured interview with organisation leaders as they are in the best position to know the extent to which supports are present within their organisations. It is comprised of 23 questions inviting respondents to describe whether, and to what degree, a range of supports are in place within their organisations to facilitate research use. Responses to each question are later scored on a three-point scale. Typically, only one leader from each organisation is required to complete ORACLe.

Unlike other measures of organisational capacity, ORACLe has a scoring system that calculates total scores by assigning a different weight to each organisational capacity domain based on its relative importance. The scoring system calculates context-appropriate total scores, and can inform organisations as to areas where they could enhance their research use capacity.

In this paper, we aim to (1) describe how the ORACLe interview was developed; (2) describe how the ORACLe interview is scored; and (3) outline how a discrete choice experiment (DCE) was used to generate the system to score ORACLe. DCEs have been applied in the area of health economics to understand patients’ preferences for different healthcare services [36-43], and can be used to determine not only what products or objects individuals prefer, but also the product/object attributes that drive these preferences and their relative importance [41,43].

## Methods

### Ethics

Ethics approval was granted by the University of Western Sydney Human Research Ethics Committee HREC Approval H9413 and H9431. Written consent was obtained from all potential respondents prior to their participation in the study.

### Development of the ORACLe interview

We aimed to develop a measure that would determine the capacity of an organisation to support and use evidence in developing policy through a number of key domains, linked to the SPIRIT action framework. A combination of strategies was used to generate the items within these domains for the ORACLe interview and each is described in detail below.

### Review of the literature

We conducted a search for articles in the area of knowledge translation, particularly those focusing on organisational barriers and facilitators to research use on SCOPUS using search terms such as ‘Research’ or ‘Evidence’, combined with ‘Health Policy’. Abstracts were examined to determine if articles were relevant, and referred to organisational barriers or facilitators to some extent. Each relevant paper was read thoroughly by two of the authors (TT and SM) to identify a wide range of concrete examples of possible tools and supports within organisations that can enhance the capacity of staff to effectively engage with research. The analysis included examples and items used in existing instruments (e.g. [23,32]). These tools and supports were then categorised into a smaller number of key domains of organisational research use capacity reflected in the SPIRIT action framework.

### Semi-structured interviews with policymakers

A purposive expert sample of (n = 9) senior Australian health policymakers were consulted to determine whether the organisational capacity domains identified in the literature were practical and applicable to real-world policy settings. Each expert was emailed a list of the organisational capacity domains identified in the literature review prior to undertaking a semi-structured interview (these domains are listed in Table 1). In the interview, experts responded to an open-ended question asking them to identify organisational strategies that facilitate the use of research in policy and program decision making. They were then asked specific questions about the relevance, applicability, and importance of each capacity domain identified in the literature search. The interviews were recorded, transcribed, and analysed to help refine the existing list of domains and identify additional tools and supports. Further details of the method and results of the interviews are described elsewhere [44].

**Table 1:** Key domains and associated examples of organisational capacity and systems to support research use in policy development identified from review of literature

Domain	Concrete examples of each domain from the literature	References
i. Documented processes to develop policy that encourage or mandate the use of research	• Organisations recognising the value and importance of evidence-informed health policymaking (for example, in the missions, vision, values, and strategic plans of the organisation) and not being resistant to change • Accessible and efficient systems, structures and processes to support and encourage research use in policy or program development – for example, templates that encourage staff to integrate research into policymaking • Incentives within the organisation to use research such as formal acknowledgement of staff by leaders or reward programs • Administrative support available for the development and implementation of research-based decisions • Recognising skills in applying research to decision-making processes in recruitment, retention, promotion, performance review, and appraisal processes within the organisation	[11, 19, 25, 27, 28, 48, 50, 63, 64]
ii. Tools and programs to assist leaders of the organisation to actively support the use of research in policy and program development	• Training workshops and programs or professional development opportunities to build leadership capacity to support use of research in policy and program development • Organisational leadership and champions of research within the organisation, with a clear vision for research use in policymaking • Incorporation of research use capacity and research skills into position descriptions, retention mechanisms, performance reviews, performance management mechanisms, and appraisals for senior policymakers • Organisational leaders disseminate research through their internal communications (e.g., newsletters, bulletins, updates, tweets, etc.) or other structured mechanisms • Tools and systems to help organisational leaders to disseminate research through their internal communications (e.g., mailing lists, tailored-targeted messages, research monitoring services, specialist staff including knowledge brokers)	[9, 11, 14, 19, 27, 28, 49, 65–68]
iii. Availability of programs to provide staff with training in using evidence from research in policy and in maintaining these skills	• Training workshops and programs for staff to improve research skills • Professional development opportunities to build research skills, or opportunities to undertake university courses • Provision of education in research • Training provided by the in-house library staff • Possessing technical capacity within the organisation to train staff to access and apply research findings to policy • Incorporating participation in training programs and development of research skills into staff performance management mechanisms, retention, and/or promotion	[11, 16, 19, 20, 24–26, 28, 48, 64, 69]
iv. Availability of supports and tools to help staff access and apply research findings	• Multifaceted access to journals, data registries, or scientific literature through subscriptions, networks, databases, intranet sites, links to research websites, and physical libraries – an infrastructure available to support staff access and use of research in policy • Availability of reference management software (e.g., EndNote) to help identify relevant research findings • Sophisticated infrastructure/systems for storing, organising, and retrieving relevant research and other resources, so they can be accessed by all staff within the organisation • Provision of an intranet site with clear links to websites that provide one-stop shopping for relevant research • Access to librarians, research experts, knowledge brokers, and clear points to gain assistance in acquiring, assessing, adapting, and applying research to policy • Knowledge intelligence services (such as electronic mailing lists, monitoring services) or staff (e.g., knowledge brokers) that scan the literature and distribute and/or communicate this throughout the organisation (e.g., through bulletins, emails, tailored targeted messages, summaries, or full articles) and other structured mechanisms to disseminate research • Availability of staff with recognised research expertise, central guidance, as well as technical, and academic support for using research	[16, 19, 24–28, 47, 48, 51, 52, 64, 65, 70–72]
v. Presence of systems/methods to generate new research evidence to inform the organisation’s work	• Organisation participates in the production of primary research, reviews, and research-derived products • Clearly defined processes, systems or units to conduct and/or commission priority research projects to inform the development of policy (e.g., a rapid response unit) either internal or external to the organisation • Clearly defined research and development strategies • Availability of technical capacity (e.g., expertise) within the organisation to undertake and generate policy-relevant research internally • Systems, processes, mechanisms, resources (e.g., funding), and supports to establish interactions with and/or partnerships with external researchers to conduct projects to inform the development of policy	[8, 16, 19, 22, 24–28, 47, 51, 52, 64, 65, 70–73]
vi. Clear methods to ensure adequate, evidence-informed evaluations of the organisations’ policies and programs	• Embedding a culture of evaluation within the organisation • Clearly defined processes and systems to conduct and/or commission evaluations of policies and programs • Availability of or access to internal or external units that conduct evaluations (e.g., service evaluation groups) and clearly defined processes for commissioning evaluations • Availability of systems and standard processes or clear frameworks to support policy evaluation, as well as sufficient resources and funding to conduct high quality evaluations • Availability of mechanisms, supports, and tools that help policymakers incorporate the findings of evaluations into policies • The processes or tools that support or inform policy evaluation encourage and/or expect staff to use research.	[13, 26, 27, 69, 74]
vii. Mechanisms that help strengthen staff relationships with researchers	• Systems, processes, mechanisms, and supports to establish interactions with and/or partnerships with researchers to assist in the integration of research into policy such as: o Access to a database of researchers or other efficient ways to identify and locate researchers to obtain advice and commission projects o Formal (and contractual) partnerships with researchers to collaborate on research projects to inform policy o Consistent, formal interactions with researchers through journal clubs, roundtables, workshops, or focus groups to gain research knowledge and/or improve research skills o Adjunct appointments of staff with (other) research organisations or universities, or other regional, provincial, or national networks o Formally inviting researchers to provide expertise or advice as a policy advisor in a committee (e.g., advisory committee, advisory panel, or working group) o Informal connections to researchers or research organisations (e.g., through phone, email correspondence, one-off meetings or presentations) o Opportunities to attend one-off forums (e.g., conferences, symposia, seminars) to hear about relevant and up-to-date findings from researchers	[1, 5, 8, 11, 16, 19, 25, 27, 28, 48, 50–52, 63–65, 67, 68, 72, 73, 75–89]
viii. Analysis	• Systems in place that strategically analyse and assess the ways that research evidence is used, and how it can best be used to inform policy decisions o Auditing and feedback systems within the organisation to assess how research evidence is currently being used by staff o Knowledge Managers that strategically analyse how research evidence is being used by the organisation to make decisions and devise strategies to improve research use in decision making o Knowledge Transfer Partnerships that organise deliberative dialogues between agencies, users, and policymakers based on evidence briefs, to formulate the best ways to incorporate these evidence briefs into policy.	[48, 78, 90, 91]

### Iterative interactions with policymakers and pilot testing

Following the literature analysis and interviews with senior policymakers, an initial set of interview questions for ORACLe was compiled. Feedback was sought from a number of senior Australian policymakers and researchers in health to determine whether the questions were appropriately worded, relevant and clear. Interview questions were then modified on the basis of this feedback. To further establish the face-validity, comprehensibility and applicability of the interview items, six pilot ORACLe interviews were undertaken in three Australian health policy agencies followed by pilot testing of the SPIRIT intervention and the associated measurement tools (including ORACLe) in one policy agency. A number of minor changes to the ORACLe interview were proposed following pilot testing, including the removal of redundant questions, modification of item wording, and inclusion of additional items to address other research resources (e.g. knowledge management systems).

### Development of the ORACLe scoring system using discrete choice experiments (DCEs)

After generating the interview items, quantitative techniques were utilised to devise a scoring system for ORACLe that appropriately weighted scores on the seven domains to produce a total score. It did not make theoretical or practical sense to assign each ORACLe domain an equal score because certain domains are likely to be more critical than others at enhancing the capacity of staff to use research in policymaking. To obtain appropriate numeric weights for each of the seven domains, we elicited the opinions of experts in health research and policy (n *=* 24) through the use of a DCE [43,45] (described in detail in elsewhere [46]). Prior to recruiting the expert sample, the DCE was pilot-tested to ensure it was optimal in terms of completion time, comprehensibility and appropriateness. Experts were then recruited by contacting corresponding authors of articles identified in the literature review described above. Snowballing was then used to expand this pool of participants by asking the identified experts to nominate others with similar knowledge and expertise. We sought the input of experts because they are highly knowledgeable about the policy context as well as the organisational barriers and facilitators to research use. Consequently, they can provide objective and context-sensitive judgments regarding the relative importance of different organisational capacity domains.

In a typical DCE, respondents do not rate individual domains (which are the attributes), but instead select between pairs of attribute combinations called profiles [41]. In our study, respondents were shown two profiles of organisations with different domain combinations and selected which organisation made the most effective use of research evidence for policy decisions. This is an ecologically valid approach because different organisations often possess multiple domains, but in different combinations (e.g. one organisation may have excellent tools to support leaders, and tools to support training in research use, but have few systems to facilitate staff capacity to access research) and they should be evaluated as such.

Following regression analyses of respondents’ choices, a utility value was calculated for each attribute (or more specifically, the particular level of each attribute), indicating the effect of that attribute on respondents’ preferences. By applying the discrete choice approach in our study, we were able to establish experts’ preferences regarding which capacity domains represented more important organisational tools and supports to facilitate research use, and obtain utilities for each capacity domain in order to calculate total scores.

### Choice of attributes (domains) and levels, experimental design

**Fig. 1:**
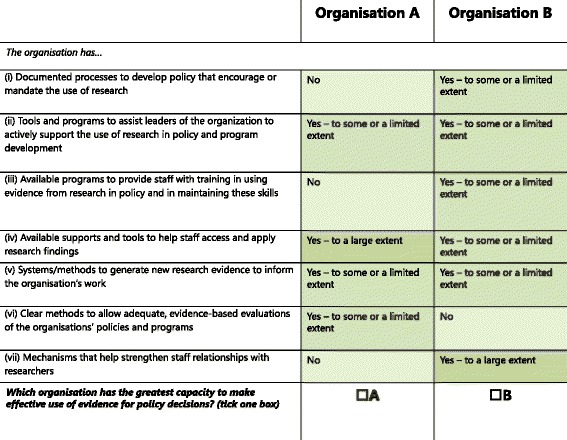
**Example of two organisational profiles in a choice pair.** Respondents are required to select which organisation makes the best use of research in policy decisions

The attributes that were used to create each profile were domains 1–7 listed in Table 1. From this point onwards, we will refer to the attributes as domains. Each domain consisted of three levels: (1) it was present to a large degree within the organisation; (2) it was present to some or a limited extent; (3) it was not present (Table 2). A series of hypothetical profiles were generated using an Orthogonal Main Effects Plan (OMEP) [43]. This method generates a series of orthogonal and balanced profiles, which allows the estimation of utility values for each domain level (i.e. main effects), but makes no provisions for the estimation of interactions [41]. Each profile represented an organisation that contained a combination of levels from all seven domains (Fig. 1). The OMEP was used to generate three sets or versions of eight pairs of profiles. In the first version, each domain consisted of only levels (1) or (2). In the second version, each domain consisted of levels (1) and (3), whereas in the third set, each domain consisted of levels (2) or (3). This produced three sets of eight profile pairs, generating 24 pairs of profiles. Participants were randomly assigned to one version of eight profile pairs.

**Table 2:** ORACLe Interview Questions and Marking Guide

Interview Question	Domain addressed	Marking Guide		
		Yes very much so	Some or to a limited extent	No
1. Does your organisation have documented processes for how policies should be developed?	Domain 1: Documented processes to develop policy that encourage or mandate the use of research	There are standard, written guidance that describe how policies should be developed and these are organisation-specific.	There are documented processes for some aspects of policy development but not all, not at a very high level, and with little detail.	There are no documented processes.
2. Do these processes encourage or require staff to use research in policy development?	Domain 1: Documented processes to develop policy that encourage or mandate the use of research	The requirement to use research must be explicitly and unequivocally noted in agency documentation (either as a requirement or encouraged), and must include *how* to use research as well as a requirement that it should be used.	Research use is **implied** but not explicitly encouraged or required in relevant documentation or if the documentation does not include *how* research should be used.	N/A if no above.
				No if there are documented processes but they do not refer to the use of research. Or, if there is a ‘culture’ or assumption of research use but it is not mandated or encouraged in relevant documentation.
			It is *implied,* if it refers to “supporting evidence” in general, rather than research evidence specifically.	
3. Are programs available for leaders to improve their confidence or expertise in use of research in policy-making?	Domain 2: Tools and programs to assist leaders of the organisation to actively support the use of research in policy and program development	*Must* specifically target leaders (rather than programs for all staff, including leaders).	Programs for everyone but includes leaders.	There are no programs that leaders would attend or that are specifically for leaders.
(Leaders mean any level of executive or management, or anyone else with a formal or informal leadership role.)				
		These programs should be offered regularly, that is, at least once a year.	OR Has one-off or occasional programs for leaders only.	
4. Do the position descriptions or performance management systems for senior policy makers in your organisation cover expertise in use of research in policy-making?	Domain 2: Tools and programs to assist leaders of the organisation to actively support the use of research in policy and program development	The expertise in use of research must be explicit and in most senior policy-makers’ PDs or similar. (policy makers not senior staff generally)	The position descriptions of senior policy makers – might refer to expertise that implies using research but is not explicit.	There is no reference to the use of research in position descriptions of senior policy makers.
			OR the expertise in use of research, although explicit, is present in only a few of the senior policymakers’ PDs	
5. In the last 6 months, have leaders of your organisation referred to research in their internal communication (e.g. newsletters, bulletins, updates, tweets, etc.)?	Domain 2: Tools and programs to assist leaders of the organisation to actively support the use of research in policy and program development	This should happen at least once a month and have happened within the last 6 months.	Referral to research in internal communications is irregular and infrequent. It happens less than once a month.	There are no relevant internal communications or if there are, leaders either do not refer to research in them or have not done so in the last 6 months.
			OR the newsletters or communications are at least monthly, but only refer to research inconsistently (less than once a month) OR SPORADICALLY	
6. Does your organisation provide access to training for staff in how to access research, appraise and apply research for policy development/implementation/evaluation?	Domain 3: Availability of programs to provide staff with training in using evidence from research in policy and in maintaining these skills	The training should be research skills specific not just referred to in the course of other training. The organisation needs to provide training internally, or allow staff to attend external training. Access to programs is actively offered to most staff, not just on an on-request basis.	Yes training is available if people say they need it – but it is not generally offered or supported, and not on an ongoing basis. OR	There is no training provided internally, and there is no support for staff to attend courses externally.
			Yes but staff may not be aware of it.	
			There is training on how to access, appraise, and/or apply research in general, not specifically for the purpose of policymaking	
		Should be regular, that is a least once a year, and available to everyone.		
7. Is participation in training on how to access research, appraise and apply research for policy development, implementation, or evaluation considered in staff performance management	Domain 3: Availability of programs to provide staff with training in using evidence from research in policy and in maintaining these skills	Staff performance management must explicitly mention training in research use or evaluation for most relevant staff.	Performance management covers only one or two of these areas, for example, applying research is not included, or evaluation is not included.	Participation in training is not considered in performance management.
			OR	
			This is only considered relevant to the performance management of a very small group of staff e.g., people whose entire job is in evaluation but not regular policy makers.	
			OR it is implied in the performance management, but not explicitly stated	
			OR it is considered as an issue, only if it has come up as an issue to have these research skills.	
8. In the last 6 months, has relevant research (papers, reports, syntheses or summary bulletins) been disseminated within your organisation?	Domain 4: Availability of supports and tools to help staff access and apply research findings	This should happen frequently, that is at least several times a month, and must have happened in the last 6 months.	This happens less than twice a month.	Relevant research has not been disseminated in the last 6 months or not at all.
		It does not matter who sends these around, i.e. colleagues on an ad hoc basis or a more systematic approach.		
9. Does your organisation have resources that provide guidance on how to access, appraise and apply research?	Domain 4: Availability of supports and tools to help staff access and apply research findings	The organisation must have documentary resources (handbooks, guidelines, online learning modules, etc.) on all three and readily available to staff.	There are limited resources or they do not cover all three aspects of research use.	There are no documentary resources.
10. Does your organisation have staff with recognised expertise in accessing, appraising and applying research to policy development/implementation/evaluation?	Domain 4: Availability of supports and tools to help staff access and apply research findings	This expertise needs to be accessible by most staff, high level and tied to a particular role rather than a person serendipitously having these skills.	This expertise is not tied to a role. Some people may have these skills but it is serendipitous and/or other staff are not generally able to access their expertise.	No – no one is available.
11. Does your organisation have research resources such as	Domain 4: Availability of supports and tools to help staff access and apply research findings	i. Topic specific journals – yes, access to all or most relevant journals is available. – This access must be provided by the organisation and not from a university login ii. For example, Medline, Embase, PsycInfo, etc. as relevant iii. A library that provides access to a range of resources, not just a shared filing system, and research is quickly and easily available through an electronic/online database (similar to how a university library works) iv. Yes to Endnote or something similar (including access if they ask for it) (This refers to the organisation providing access, not to access through other means e.g., staff member’s university affiliation).	i. Yes some journal access but cannot access many of the journals needed. ii. Yes access to some databases or a database but several key databases are not available iii. Yes but it takes a long time to get access to full text articles, or can’t get full text, or yes but doesn’t stock many of the key books required iv. No midpoint here as you would not require more than one	i. No journal subscriptions ii. No subscriptions to databases iii. No access to a library or electronic library iv. No licenses for reference management software
i. Subscriptions to research journals? (e.g. …) ii. Subscriptions to databases of research publications? iii. A library or an electronic library? iv. Licenses for reference management software (e.g. Endnote)				
*Note: these questions refer to resources provided by the organisation, and does NOT include resources owned by individual employees (such as university logins, their own licenses to EndNote). If this is the case for any of these, score 1.*				
12. Does your organisation have established methods for commissioning reviews of existing research?	Domain 4: Availability of supports and tools to help staff access and apply research findings	Yes there is a standard written process which staff are expected to use when commissioning research.	Yes but the methods are verbal/ad hoc/situation by situation.	There are no methods for commissioning reviews OR the organisation does not commission reviews of existing research.
		If the Sax E-check is used, or any other organisation’s or institution’s rapid review process, then that would be scored in this category		
13. Does your organisation have systems for managing knowledge from research? (e.g. systems for retrieving, collating, storing and translating external and internal research)	Domain 4: Availability of supports and tools to help staff access and apply research findings	There are shared filing systems, databases, etc. that are easily searchable and accessible by most relevant staff.	This is kept in one place and accessible but not indexed or easily searchable. The organisation relies on corporate memory to know what research has been done and where it is.	There is no central storage place and no process for managing knowledge from research.
		Needs to be well-organized and structured; not simply a big folder or drive where the whole range of files (including non-research related documents)		
			Centralised system but disorganized, or not completely developed yet. TRIM is one example, unless they have highly organised it.	
14. In the last 6 months, has your organisation undertaken internal research to support policy development/implementation/evaluation?	Domain 5: Presence of systems/methods to generate new research evidence to inform the organisation’s work	Must have been in the last 6 months and undertaken by staff of the organisation. It includes at least one large or in-depth piece of internal research, or several smaller pieces of internal research.	One small pieces of basic internal research.	No never or not in the last 6 months.
(For example focus groups, satisfaction surveys), but not evidence check.)				
*This question does NOT include whether the organisation has undertaken evaluations of their policies. This is captured in Qs 16-18*				
15. In the last 6 months, has your organisation commissioned external research to support policy development/implementation/evaluation?	Domain 5: Presence of systems/methods to generate new research evidence to inform the organisation’s work	Research undertaken by another organisation (potentially in partnership with the organisation Must have been in the last 6 months and more than once.	In the last 6 months but only once	No never or not in the last 6 months.
***External research to inform policy development Or to inform the implementation or evaluation of a policy or program. This question is not about whether the organisation evaluates their policies.***				
16. Does your organisation encourage or require that evaluation be built into policy development and program planning?	Domain 6: Clear methods to allow adequate, evidence-informed evaluations of the organisations’ policies and programs	There is an explicitly documented organisational requirement that evaluation be built into every policy/program.	Yes this is expected but not required, or is not required of all programs	Evaluations do not occur or occur occasionally but there is no organisational requirement to conduct them.
(Questions 16–18 include situations where evaluations are commissioned externally).			OR is just about to be rolled out (and so is happening partially, over some programs and policies)	
17. Does your organisation have documented processes for how policies should be evaluated?	Domain 6: Clear methods to allow adequate, evidence-informed evaluations of the organisations’ policies and programs	The processes must explain in detail how the policies should be evaluated.	Yes there are documented processes which are very general.	No documented processes
			OR documented processes are developed on a case by case basis or following initial preparations	
18. Do these processes encourage or require staff to use research in policy evaluation OR are these evaluation processes and methods based on research?	Domain 6: Clear methods to allow adequate, evidence-informed evaluations of the organisations’ policies and programs	The requirement to use research must be explicit and unequivocal.	The processes refer to research but do not encourage or require that research be used.	There are documented processes but there is no requirement to use research or there are no documented processes regarding evaluation.
			*Or the evaluation is conducted by an expert who we assume has been influenced by research in their approach (more indirect use of research)*	
*NOTE: this does NOT refer to collecting data as part of the evaluation. This is about whether the evaluation approach used by the organisation is based on research, or requires staff to use research to guide the evaluation*		(This might include researching evaluation methods as well as scene setting). “Research” does not include data collection whereas question 14 does.		
		*Either the guidelines are research-evidence based, or the guidelines instruct the individual to search for research evidence to support their evaluation approach.*		
*NOTE: If answered NO to question 17, then score 1 for this question, even if the evaluation is performed by an expert.*				
19. In the last 6 months, has your organisation been represented at any research forums or conferences?	Domain 7: Mechanisms that help strengthen staff relationships with researchers	Attendance at such events was common and by a range of staff.	Only a certain level of policy maker attends, or only attends as invited speakers, or only attends rarely.	No not in the last 6 months or not at all
20. Does your organisation have formal, contractual relationships with external research organisations?	Domain 7: Mechanisms that help strengthen staff relationships with researchers	Any formally documented relationship counts. Short term relationships are fine, if these are active at the time of the interview. There need to be several such relationships and a sense that these (or others) were likely to continue, and that having such relationships was important to their ongoing work.	Just one currently.	No, this does not happen or there are none currently.
21. Does your organisation have informal, collaborative relationships with external research organisations?	Domain 7: Mechanisms that help strengthen staff relationships with researchers	Any un-formalised relationship (including on a staff to staff basis) counts here.	Just one currently.	No, this does not happen or there are none currently.
22. Do members of your organisation have joint or adjunct appointments in research organisations?	Domain 7: Mechanisms that help strengthen staff relationships with researchers	Usual examples would be adjunct appointments in universities. A high rating would mean that several staff had such positions. This also includes where staff work part time at the agency in question and are also employed by a research organisation.	Just one currently.	No, this does not happen or there are none currently.
23. In the last 6 months, have researchers participated in policy advisory committees (or similar) in your organisation?	Domain 7: Mechanisms that help strengthen staff relationships with researchers	The involvement for researchers in these types of roles is frequent, that is, it happens more than once in 6 months, and is systematic (not serendipitous).	Only once in the last 6 months.	No this does not happen or has not happened in the last 6 months

The OMEP was also used to generate a common set of eight profile pairs. In this set, the domains contrasted level (1) (i.e. the attribute was not present) with level (3) (i.e. it was present to a large extent). Altogether, each respondent was asked to evaluate 16 pairs of profiles: eight profile pairs came from one of the three versions described above, and the other eight profiles pairs came from the common set. For the DCE itself, participants were exposed to each profile pair through an online survey. The profile pair consisted of two organisations that contained different combinations of levels of each of the seven domains. They were required to select which organisation in the pair made more effective use of research evidence in policy decisions (see Fig. 1 for an example).

### Model calculation

After respondents made their choices, a conditional logit model was estimated from the combination of the common and master designs. A quadratic, as opposed to a linear relationship between domain levels and choice was used in the model calculation as it better represented the data. Consequently, each domain has a regression coefficient for its linear and quadratic component. These regression coefficients were used to calculate importance values to determine which domains had the strongest impact on respondents’ choices.

## Results

### The ORACLe interview

Eight domains of organisational capacity were identified from the literature review: (1) documented processes to develop policy that encourage or mandate the use of research; (2) tools and programs to assist leaders of the organisation to actively support the use of research in policy and program development; (3) availability of programs to provide staff with training in using evidence from research in policy and in maintaining these skills; (4) availability of support and tools to help staff access and apply research findings; (5) presence of systems/methods to generate new research evidence to inform the organisation’s work; (6) clear methods to ensure adequate evidence-informed evaluations of the organisations’ policies and programs; (7) mechanisms that help strengthen staff relationships with researchers; and (8) systems that analyse the ways that research can inform policies and programs. Table 1 lists each domain and concrete examples of each domain identified from the literature search.

The semi-structured interviews with senior policymakers verified the applicability and appropriateness of domains 1–7. However, there was a lack of consensus regarding the definition of domain 8 (systems in place to analyse the ways research can better inform policies and programs), with only two interviewees being able to provide concrete examples of this domain. Consequently, a preliminary ORACLe interview schedule was developed incorporating domains 1–7 only, and this was further refined through iterative discussions with senior policymakers and pilot testing. The final ORACLe interview schedule contains 23 questions which address capacity domains 1–7 as displayed in Table 1. Table 2 displays the full ORACLe interview schedule and the organisational capacity domain addressed by each question.

### The ORACLe scoring system

**Fig. 2:**
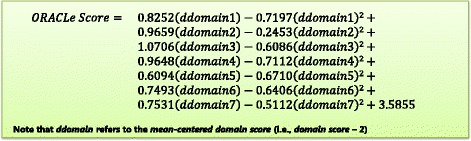
**Conditional logit model estimated from experts’ choices in the DCE.** This model is used as the basis for calculating total ORACLe scores

The DCE data was analysed and the estimated conditional logit model is displayed in Table 3 and Fig. 2. Results revealed that each domain was significantly (and positively) related to respondents’ choices. In other words, respondents preferred organisations that possessed greater amounts of each domain.

**Table 3:** Conditional logit model estimated from choices of expert respondents

Effect	Estimate	Standard error	95% Confidence interval	
Intercept	3.59^***^	0.53	2.55	4.62
a1mc^a^	0.83^***^	0.15	0.53	1.12
a2mc	0.97^***^	0.14	0.69	1.24
a3mc	1.07^***^	0.17	0.74	1.40
a4mc	0.96^***^	0.16	0.65	1.28
a5mc	0.61^***^	0.12	0.38	0.84
a6mc	0.75^***^	0.13	0.49	1.01
a7mc	0.75^***^	0.15	0.46	1.05
a1sq^b^	−0.72^**^	0.22	−1.16	−0.28
a2sq	−0.25	0.22	−0.68	0.19
a3sq	−0.61^**^	0.23	−1.06	−0.16
a4sq	−0.71^**^	0.23	−1.16	−0.26
a5sq	−0.67^**^	0.21	−1.08	−0.26
a6sq	−0.64^**^	0.22	−1.07	−0.21
a7sq	−0.51^*^	0.22	−0.95	−0.07

^*^ *P* <0.05, ^**^ *P <*0*.*01, ^***^ *P <*0*.*001

^a^The ‘mc’ suffix indicates that the domain score has been mean centred (i.e. domain score – 2)

^b^The ‘sq’ suffix indicates that the mean centred domain score has been squared

Using this model, importance values were calculated to determine which domains had the strongest impact on respondents’ choices (Table 4). Based on the results, all domains significantly contributed to respondents’ choices, although domain 3 (tools/programs to provide staff with training in using research evidence) had the largest impact, followed by domains 2 (tools to assist leaders actively support research use within the organisation) and 4 (availability of support and tools to help staff access and apply research findings).

**Table 4:** Relative importance values of each ORACLe domain

Domain	Importance
(1) Documented processes to develop policy that encourage or mandate the use of research	11.88%
(2) Tools and programs to assist leaders of the organisation to actively support the use of research in policy and program development	19.48%
(3) Availability of programs to provide staff with training in using evidence from research in policy and in maintaining these skills	20.53%
(4) Availability of support and tools to help staff access and apply research findings	17.57%
(5) Presence of systems/methods to generate new research evidence to inform the organisation’s work	8.74%
(6) Clear methods to allow adequate, evidence-informed evaluations of the organisations’ policies and programs	10.96%
(7) Mechanisms that help strengthen staff relationships with researchers	10.84%

The conditional logit model provides the basis for calculating total ORACLe scores. The model weight each capacity domain differently, based on the experts’ opinions regarding which domains are most important to strengthening organisations’ capacity to use research in policy. The steps involved in calculating ORACLe total scores are described below.

### Using the interview and model to score ORACLe

ORACLe should be scored by an objective coder who was not directly involved in interviewing respondents. Preferably, coders should have experience in psychometric measurement or rating observable behaviour, but not necessarily be experts in knowledge translation or implementation science. To score ORACLe, objective coders examine the responses given to each of the 23 questions administered in interviews with organisational leaders (using interview transcripts and audio recordings) and assign a score indicating the extent to which each support is present within the organisation using the following three-point scale: (1) The tool/support is present to a large extent (score of 3), (2) The tool/support is present to some or a limited extent (score of 2), or (3) the tool/support is not present at all (score of 1). Scoring of each item is guided by a marking guide, which provides detailed descriptions of each of the abovementioned scoring categories (Table 2). The full interview and marking guidelines for each question is displayed in Table 2. Experience to date suggests that the ORACLe scores assigned by independent raters using the scoring guide exhibit a high level of inter-rater agreement [46].

**Fig. 3:**
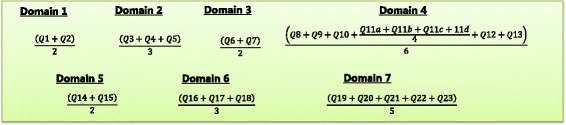
**Domain score formulae.** These are the formulae to calculate scores for each of the seven ORACLe domains (i.e. domain scores)

After scoring each individual question, scores for questions within the same domain are averaged to produce a domain score, one for each of the seven domains (Fig. 3). These seven domain scores are then mean-centred (i.e. subtract 2 from each domain score) and substituted into the equation in Fig. 2 to compute the total ORACLe score which ranges from 0 to 10. This represents the score assigned to that particular agency, where higher scores are indicative of greater tools and support within the organisation to support research use in policy. These steps are outlined in Additional file 1, which provides SPSS syntax to calculate total ORACLe scores from raw data entered into an the data frame provided in Additional file 2.

## Discussion

This paper describes the development of a comprehensive, theory-based, structured interview measure of an organisation’s capacity to engage with and use research in policymaking, named ORACLe, and the establishment of a scoring system for the measure. A multifaceted approach was used to generate an interview schedule that encompassed a vast range of tools and systems that organisations may have in place to enhance staff capacity to engage with and use research. Further, a DCE was used to generate a scoring system for ORACLe that assigns appropriate weights to each ORACLe domain in the calculation of total scores, based on expert opinion regarding the most important strategies to support research use capacity within organisations. Our research thus represents the first attempt to empirically quantify the relative importance of different organisational structures and supports, and use this information to generate a valid system to score organisations’ research use capacity.

From the DCE results, domain 3 – tools/programs to provide staff with training in using research evidence – yielded the largest importance value. Therefore, our expert sample regarded the provision of staff training in accessing and applying research to policy as the most important component of an organisations’ research use capacity. This result parallels those in other sectors, including Meijers et al.’s systematic review [47], where research use among nurses was strongly associated with the degree of multifaceted access to research resources and support, and the provision of training in research use by the organisation.

Domain 2 – tools to assist leaders actively support research use within the organisation – had the next largest importance value. Thus, the expert sample placed a great deal of importance in organisations providing programs to support research use leadership. Helmsley-Brown [28] emphasised that leaders were critical to establishing an evidence-based organisational culture and direction that encouraged and motivated reflection, criticism of existing practices, free expression of experiences, and use of research. Gold [27] echoed this perspective, stating that leadership was important in allaying employees’ concerns about using research and promoting a mutual understanding between policymakers and researchers. In support of these views and our findings, El-Jardali et al. [48] found that strong organisational leadership was a key factor in promoting evidence-informed policy initiatives (e.g. programs to improve research use capacity among decision makers); establishing collaborative partnerships between researchers, users, and funders; increasing awareness of the value of research use in policy; and bringing about greater use of research in policymaking. One currently available leadership program is EXTRA (Executive Training for Research Application), which aims to increase the capacity of health service executives to access, promote, and use research, as well as to increase their organisation’s receptivity to research use [49]. Evidence indicates that the EXTRA program leads to self-reported improvements in research literacy (e.g. accessing and conducting research) and skills in promoting use of research evidence within the organisation and modest changes in staff receptivity to research.

Domain 4 – the availability of supports and tools to help staff access and apply research also emerged as a relatively important domain of organisational research use capacity. This finding coincides with numerous studies emphasising the importance of providing staff with resources to assist research access and use. For example, Canadian policymakers and research specialists stated that the most important element impacting upon research use was the organisational climate, and in particular, the availability of infrastructure and specialist staff to help policymakers use and apply research evidence [19]. Similarly, Evans et al. [26] found in their interviews with policymakers, that the availability of central guidance, technical and academic support, supportive resources, and standardized frameworks to conducting research and evaluation, were positively related to policymakers’ capacity to use research and conduct rigorous evaluations of policies.

In contrast to these results, the lowest importance value emerged for domain 5 – presence of methods to generate new research to inform the organisation’s work. Therefore, relative to the domains described above, our experts did not regard the presence of processes to conduct or commission data analyses, research studies or evaluation as particularly important to building an organisations’ capacity to engage with and apply research to policy. This is most likely due to the overwhelming emphasis on increasing organisations’ capacity to engage with existing research findings as opposed to producing new research to inform policy and that, in general, policy agencies are not in the business of doing research [19,50].

The expert sample also regarded domain 7 – mechanisms that help strengthen staff relationships with researchers – as less important in contributing to overall capacity of the organisation to use research in policy, relative to the other domains. This is unexpected given the preponderance of existing evidence emphasising the importance of organisations establishing ongoing partnerships with researchers and developing mechanisms to allow such contact to be established [16,18,51,52]. In light of their expertise in both policy and research, the expert sample may have felt that if policymakers had developed research skills themselves through training programs and professional development opportunities (i.e. domain 2), there might be less of a need to consult researchers. Furthermore, the experts may have felt that policymakers would be more likely to gain a balanced perspective on policy issues by evaluating the research themselves, versus consulting researchers who may have fixed opinions on those issues [53,54]. These explanations, however, are only conjecture and further qualitative research is required to understand why the experts prioritised particular domains over others.

ORACLe has been developed to overcome many of the limitations of previous measures described in the introduction [20,32,47]. Firstly, it specifically addresses organisational tools and capacity to use research in policymaking, as opposed to measuring organisational culture/capacity very generally. Secondly, ORACLe is a theory-based measure, grounded in the SPIRIT Action Framework, and therefore emphasises that organisational research capacity and culture is critical to promoting staff capacity to engage with and use research, and enabling the development of evidence-informed policies. Third, it is a comprehensive measure, assessing seven domains of organisational capacity and culture which expands upon previous organisational capacity measures (e.g. [32]). Fourth, because ORACLe is completed by organisational leaders, it directly assesses the availability of tools and supports to encourage research use, as opposed to measuring staff perceptions of these supports and tools [23,32,47]. Leaders are the most reliable respondents in this context since they would have been responsible for putting in place these research use tools and supports within their respective organisations. This is a key advantage of ORACLe over previous measures of organisational research use capacity. Agencies, however, often have multiple divisions led by different senior staff. Consequently, a single executive might not be aware of all the tools and systems in place across the agency. One approach, as utilised by Kothari et al. [32], would be to gather a small representative group (four to six) of senior staff from each organisation to complete ORACLe to ensure a more valid and objective assessment of the agency’s systems and culture. This kind of inter-rater reliability should ideally be incorporated into future testing and use of the measure.

Fifth, one of the major strengths of ORACLe is its empirically derived scoring system. By conducting a DCE with an expert sample, we were able to generate a model to calculate total scores on ORACLe. In this calculation, the model assigns different weights to each of the seven domains based on experts’ opinions regarding which domains are most important to strengthening organisations’ capacity to use research in policy. As a result, it allows users to calculate total scores on ORACLe that are context-sensitive, appropriate, and in line with experts’ preferences. This represents a major advance on previous tools which were unable to weigh different aspects of organisational research use capacity based on their importance.

Another key advantage of the ORACLe scoring system is that it allows organisations to identify their specific capacity development needs, and the relative importance of these capacities. Organisations can use their scores on each domain to guide decisions about which tools and supports to invest in to improve their research use capacity. For example, if an organisation scores low on domain 3, its leaders may choose to invest in a range of training programs to help staff better engage with and use research in policy. Furthermore, given that domain 3 yielded the highest importance value, investments into improving this capacity may be more likely to yield considerable improvements in the organisations’ overall research use capacity.

In terms of limitations, our sample size was relatively small compared to other DCE studies [55]. Hence, it is possible that the model underlying our scoring system is specific to our particular sample of experts, and that a different model would emerge in another, larger sample. A review of choice studies conducted in the health field revealed that the average sample was 259, and ranged from 13 to 1258 [56]. Sample sizes should be at least 150 if the target population is very large, although this would not be the case for the population of knowledge translation experts [55]. Orme [55] recommended that, when conducting pilot work or developing hypotheses about a particular target group, 30–60 participants may be sufficient. Our current sample approaches this cut-off.

Despite the relatively small sample size, we are confident in our findings for three main reasons. Firstly, our sample consisted of a diverse group of experienced knowledge translation experts. Secondly, supplementary analyses revealed that there were no statistically significant differences between experts in their choice preferences (see [46] for details). Thirdly, we have no reason to believe that the DCE survey was overly complex for respondents and invalidly elicited their preferences, since the number of domains was kept low (i.e. seven), and all domains and levels were clearly presented, concretely defined, and operationally distinct [41].

ORACLe aims to measure a range of domains in order to inform agencies of opportunities to improve policymakers’ use of research. It is important to note, however, that organisational change is highly complex, and that top-down initiatives alone are unlikely to bring about changes in staff capacity and attitudes towards research use [57]. Recognition of the wider organisational system is essential. Many studies emphasise the importance of social processes, including the role of networks and key change agents (e.g. champions and opinion leaders), in facilitating the uptake of innovations [58,59]. Further, the concept of ‘good practice’ and how different types of knowledge are valued are often shaped by collective responses to local circumstances which may demand local (rather than standardised) solutions [58]. These social and contextual influences are not explicitly addressed in ORACLe (as they are less amenable to quantitative measurement and intervention), but are nonetheless critical considerations when designing and implementing initiatives for building organisational capacity in using research.

ORACLe has yet to be validated as a measure of organisational capacity and culture to support research use. Validity testing may involve investigating its factorial structure, and verifying whether items load onto the seven organisational domains obtained in Table 1 as well as a common organisational capacity factor. However, given that ORACLe is completed by agency executives, it would take time to recruit a sufficiently large sample to undertake factor analysis. We considered the possibility of inviting non-senior staff (i.e. policymakers) to do ORACLe as a strategy to increase sample size. It is likely that staff would have different perceptions regarding their organisation’s systems and tools, relative to executives. For example, staff might not be aware of all the tools available within their organisation. However, ORACLe was designed to be an objective (and thus, valid) measure of whether a range of systems and tools exist within the organisation to support research use, as opposed to staff perceptions (or awareness) of these systems and tools. Hence, ORACLe targets informants who would have first-hand knowledge of this information, which, for the most part, would be the executive staff. The need for a knowledgeable informant necessarily places limitations on sample size, and means that ORACLe is a tool more suited to formative than summative purposes. We have developed a companion tool, SEER [35], which includes scales measuring staff perceptions/awareness of organisational systems and support. Measurement of perceptions is important, since a lack of awareness of organisational tools and systems among staff is likely to negatively impact upon their use of research in policy [19]. We plan to examine convergence of responses on ORACLe and SEER as part of further tests of the validity of both measures (see below).

A practical approach to validity testing would be to test whether ORACLe scores are predictive of relevant outcomes such as policymakers’ skills and values towards research use, and their use of research in policymaking. Presumably, if organisations score well on ORACLe, then policymakers should hold more positive attitudes to research use, exhibit greater engagement with research (e.g. actively searching for and appraising research), participate in more active collaborations with researchers, and show greater use of research in policymaking. SEER [35] is one such measure that evaluates policymakers’ attitudes and skills regarding research use, as well as their engagement with, and use of research in their work. Another measure we have developed, SAGE [60-62], measures the extent to which research was engaged with and used in discrete policy or program documents. We are currently testing the convergent validity between ORACLe and these two measures. Further validity testing might involve examining whether ORACLe is predictive of more distal outcomes such as financial expenditures and health outcomes.

## Conclusions

In this paper, we have described the qualitative and quantitative development of a system to measure and score organisations’ capacity to engage with and apply research to policymaking. The qualitative development ensured that the measure was thorough, face-valid, and captured all the main elements of organisational capacity. The quantitative development produced a scoring system that not only assigns context-appropriate total scores, but can inform organisations about what capacities and tools require further development and investment, and the relative importance of these capacities. We hope that the development of this measure will trigger initiatives to improve organisations’ tools and support, increase research capacity among staff, and drive the ongoing development of evidence-informed health policies.

## Additional files

Additional file 1: **SPSS Instructions and syntax.** These are the instructions and SPSS syntax to calculate domain scores and total scores for ORACLe. (DOCX 29 kb)

Additional file 2: **Data frame.** This file provides the data frame in .csv format, to enter raw data following scoring of the ORACLe interview. (CSV 162 bytes)
