# Supplementary material for: The development of ORACLe: a measure of an organisation’s capacity to engage in evidence-informed health policy
Source: Health Res Policy Syst. 2016 Jan 14;14:4. doi: 10.1186/s12961-015-0069-9 (PMC4712550; doi:10.1186/s12961-015-0069-9)
Supplement: Supplementary file 1 — SPSS Instructions and syntax. These are the instructions and SPSS syntax to calculate domain scores and total scores for ORACLe. (DOCX 29 kb) [file 12961_2015_69_MOESM1_ESM.docx]

**Supplementary File 1**

Below is the syntax for calculating total ORACLe scores. The syntax below assumes you have entered scores for questions 1-23 on 23 separate variables (columns), and one line per respondent. Supplementary File 2 contains the data frame (in .csv format) that you can use to enter data for individual respondents.

Steps:

1. Open the data frame (ORACLE.csv) into SPSS (choose “text” in the Files of Type tab) by navigating to where you saved the file on your computer. Supplementary File 2 is the data frame file you will need.
   1. Follow through the import steps.
      1. Step 1: Click Next
      2. Step 2: Choose *Delimited* for how variables are arranged
         Choose “YES” for variable names included at the top of the file
      3. Step 3: Click Next
      4. Step 4: Un-tick “space” for which delimiters appear between variables. Ensure “Comma” is still ticked
      5. Step 5: Click Next
      6. Step 6: Click Finish (unless you wish to save the file format for future use, or paste the steps into a syntax file)
2. Once you have opened the data frame, enter the data for questions 1-23 for each respondent into the data frame.
3. Paste the syntax below into the SPSS syntax editor.
4. Highlight the syntax and click RUN it to produce the total scores. Total scores will appear on their own separate variable called “TOTAL” in the data file.

#calculate domain scores.

COMPUTE DOMAIN1=(Q1 + Q2)/2.

EXECUTE.

COMPUTE DOMAIN2=(Q3 + Q4 + Q5)/3.

EXECUTE.

COMPUTE DOMAIN3=(Q6 + Q7)/2.

EXECUTE.

COMPUTE Q11=(Q11A + Q11B + Q11C + Q11D)/4.

EXECUTE.

COMPUTE DOMAIN4=(Q8 + Q9 + Q10 + Q11 + Q12 + Q13)/6.

EXECUTE.

COMPUTE DOMAIN5=(Q14 + Q15)/2.

EXECUTE.

COMPUTE DOMAIN6=(Q16 + Q17 + Q18)/3.

EXECUTE.

COMPUTE DOMAIN7=(Q19 + Q20 + Q21 + Q22 + Q23)/5.

EXECUTE.

#calculate mean centred domain scores.

COMPUTE DDOMAIN1=DOMAIN1 - 2.

EXECUTE.

COMPUTE DDOMAIN2=DOMAIN2 - 2.

EXECUTE.

COMPUTE DDOMAIN3=DOMAIN3 - 2.

EXECUTE.

COMPUTE DDOMAIN4=DOMAIN4 - 2.

EXECUTE.

COMPUTE DDOMAIN5=DOMAIN5 - 2.

EXECUTE.

COMPUTE DDOMAIN6=DOMAIN6 - 2.

EXECUTE.

COMPUTE DDOMAIN7=DOMAIN7 - 2.

EXECUTE.

#compute unadjusted total score.

COMPUTE TOTAL=3.585484 + 0.825159*DDOMAIN1 - 0.719588*((DDOMAIN1)**2) + 0.965865*DDOMAIN2 - 0.245269*((DDOMAIN2)**2) + 1.070595*DDOMAIN3 - 0.608552*((DDOMAIN3)**2) + 0.964807*DDOMAIN4 - 0.711235*((DDOMAIN4)**2) + 0.609399*DDOMAIN5 - 0.671005*((DDOMAIN5)**2) + 0.749341*DDOMAIN6 - 0.640577*((DDOMAIN6)**2) + 0.753117*DDOMAIN7 - 0.511225*((DDOMAIN7)**2).

EXECUTE.

#compute adjusted total score out of 9 (minimum 0, maximum 9).

COMPUTE ADJUSTED_TOTAL=(TOTAL + 6.46025)*0.757794803.

EXECUTE.
